# RESILIENCE AND SOCIAL CONNECTION AMONG MARGINALIZED OLDER ADULTS: CHALLENGES AND OPPORTUNITIES

**DOI:** 10.1093/geroni/igae098.0096

**Published:** 2024-12-31

**Authors:** Melanie Levasseur, Andrew Wister

**Affiliations:** Chair; Co-Chair

## Abstract

Resilience, i.e., the ability to cope and thrive in the face of adversity, and social connection are recognized factors of older adults’ health, well-being and community integration. Improving resilience and social connection is especially challenging in marginalized older adults, who face multiple barriers to social participation. This symposium will present first theoretical contribution followed by empirical evidence where resilience and social connection were enhanced in marginalized older adults. The presentations will provide a forum to discuss the challenges and opportunities for better supporting resilience and social connection among marginalized older adults from Canada. Levasseur and colleagues examined the associations between vulnerability and life satisfaction, and the moderating effect of social support. Wister and colleagues discuss a resilience model designed to support recovery from social isolation and loneliness, which relies on internal and external resources across a socio-ecological spectrum. Majón-Valpuesta and colleagues considered the determinants regarding social participation opportunities for older transgender baby boomers in Canada. Meynet and colleagues identified and described studies assessing the efficacy of home-based and collective interventions on older adults’ social participation, isolation, loneliness, and social connectivity. Li and colleagues verified the association of loneliness in long-term spousal caregivers with social participation and social support. Together, these presentations provide insights to understand how resilience and social connection can alleviate the risk of marginalization and promote health.

